# Annual incidence and fatality rates of notifiable infectious diseases in southeast China from 1950 to 2022 and relationship to socioeconomic development

**DOI:** 10.7189/jogh.13.04107

**Published:** 2023-09-08

**Authors:** Jianli Hu, Wei Li, Zhihang Peng, Ziying Chen, Yingying Shi, Yanze Zheng, Qi Liang, Ying Wu, Wendong Liu, Wenqi Shen, Qigang Dai, Liguo Zhu, Changjun Bao, Fengcai Zhu, Feng Chen

**Affiliations:** 1 School of Public Health, Nanjing Medical University, Nanjing, China; 2 Department of Acute Infectious Diseases Control and Prevention, Jiangsu Provincial Center for Disease Control and Prevention, Nanjing, China; 3 National Health Commission Key Laboratory of Enteric Pathogenic Microbiology, Nanjing, China; 4 General office, Jiangsu Institute of Parasitic Diseases, WuXi, China; 5 Department of Acute infectious Diseases Control and Prevention, Lianyungang Municipal Center for Disease Control and Prevention, Lianyungang, China; 6 Jiangsu Province Engineering Research Center of Health Emergency, Nanjing, China; 7 China International Cooperation Center for Environment and Human Health, Nanjing Medical University, Nanjing, China

## Abstract

**Background:**

Over the past 70 years, China has advanced significantly in the prevention and treatment of infectious diseases while simultaneously undergoing a socioeconomic transformation, making it a useful source of data for analysing relationships between public health policy and the control of infectious diseases.

**Methods:**

We collected data on the incidence of notifiable infectious diseases and associated fatalities in Jiangsu province in southeast China from the Provincial Center for Disease Control and Prevention, Provincial Institute of Parasitic Diseases, and the Nationwide Notifiable Infectious Diseases Reporting Information System. We compared data from different historical periods using descriptive statistical methods, joinpoint regression, and correlation analysis.

**Results:**

During 1950-2022, 75 754 008 cases of 46 notifiable infectious diseases were reported in Jiangsu, with an average annual incidence was 1679.49 per 100 000 population and a fatality rate of 1.82 per 1000 persons. The incidence of classes A-B decreased (average annual percent change (AAPC) = -2.1) during the entire study period, while the incidence of class C increased (AAPC = 10.8) after 2004. The incidence of intestinal diseases (AAPC = -4.4) and vector-borne and zoonotic diseases (AAPC = -8.1) decreased rapidly, while the incidence of sexually transmitted and blood-borne diseases (AAPC = 1.8) increased. The number of medical and health institutions and the per capita gross domestic product correlated negatively with the annual incidence of diseases in classes A-B, but not with fatality rates.

**Conclusions:**

Although the annual incidence of many severe infectious diseases has decreased in Jiangsu since 1950, the incidence of sexually transmitted and blood-borne diseases increased. Socioeconomic growth and sustainable investment in health systems are associated with better control of infectious diseases.

Although the global burden of disease has shifted from communicable to non-communicable illness in many countries [1], epidemics and pandemics of infectious diseases continue to impact society, as seen with the current coronavirus 2019 (COVID-19) pandemic. Since the founding of the People's Republic of China in 1949, China has made significant advances in eradicating infectious diseases either completely (such as smallpox) or in greater part (such as poliomyelitis, filariasis, leprosy, and neonatal tetanus) [2,3]. While infectious diseases shortened life expectancy in China by an average of 15.59 years in 1950, this decreased to 0.07 years in 2010 [4]. The annual incidence of 18 notifiable infectious diseases decreased rapidly from more than 4000 cases per 100 000 in 1970 to fewer than 250 per 100 000 in 2007 [5].

These public health achievements have occurred simultaneously with drastic socioeconomic changes, which can be approximately divided into four phases: rapid socioeconomic development (1949-1965), stagnation (1966-1978), rapid growth (1979-2003), and post-severe acute respiratory syndrome (SARS) (2004-2022) [2]. During these phases, national and local governments made extensive investments in healthcare infrastructure. These simultaneous processes make China a useful case study for exploring how socioeconomic development and public health policy can affect the incidence of infectious diseases and their associated fatality; studying them could help China and other countries develop and optimise public health policies and interventions in the future.

However, existing studies have focused on the incidence and fatality rates of a few individual diseases such as Japanese encephalitis [6], brucellosis [7], plague [8,9], and haemorrhagic fever with renal syndrome [10]. There is still a lack of comprehensive analyses on the evolving incidence and fatality rates of various infectious diseases over the past 70 years. We therefore aimed to analyse the dynamics of incidence and fatality of 46 infectious diseases in the southeast Chinese province of Jiangsu from 1950 to 2022, and investigate their association with socioeconomic development to provide the most comprehensive dynamic profile of infectious diseases over a long time period.

## METHODS

### Study setting

**Figure 1:**
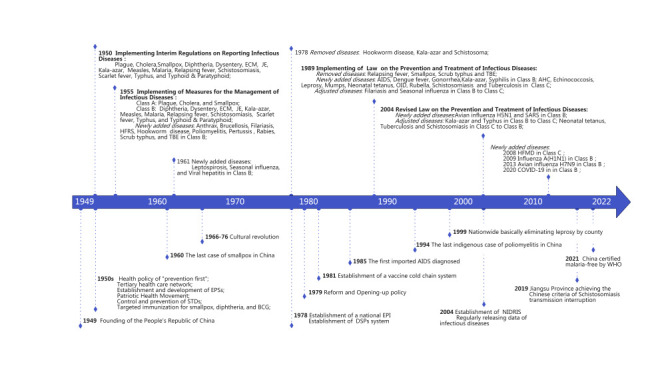
Historical events and national policies related to the prevention and control of infectious diseases in China. AHC – acute haemorrhagic conjunctivitis, AIDS – acquired immune deficiency syndrome, BCG – *Bacillus Calmette-Guerin*, DSP – disease surveillance point, ECM – epidemic cerebrospinal meningitis, EPI – Expanded Program on Immunization, EPS – epidemic prevention station, HFRS – haemorrhagic fever with renal syndrome, HFMD – hand-foot-mouth disease, JE – Japanese encephalitis, NIDRIS – Nationwide Notifiable Infectious Diseases Reporting Information System, OID – infectious diarrhoeal diseases other than cholera, dysentery, or typhoid/paratyphoid, SARS – severe acute respiratory syndrome, STDs – sexually transmitted diseases, TBE – tick-borne encephalitis.

We based our analyses on Jiangsu province, as it ranks second in per capita gross domestic product (GDP) among the 31 provinces and autonomous regions in China, with approximately 85 million inhabitants. Notably, it was the first province to use the notifiable infectious disease reporting system among Chinese provinces. Established in 1950, this system initially covered 15 infectious diseases, expanding to 35 in 1989 and 40 since 2020 [11] (**Figure 1**). Forty-six infectious diseases were defined as notifiable in China from 1950 to 2022.

### Notifiable disease data

China's notifiable infectious disease reporting system collects data from the entire population. Previously, infectious disease control case report carts were filled out when all suspected and confirmed cases of infectious diseases were diagnosed by a clinician in medical institutions. These cards were required to be initially mailed on paper to the local center for disease control and prevention (CDC), then sent in electronic form starting in 1985. The local CDC staff conducted manual statistics to form aggregated data, reporting their findings step by step. After 2004, these cards are directly entered by the personnel of medical institutions through the Nationwide Notifiable Infectious Disease Reporting Information System (NIDRIS).

We obtained aggregated data of notifiable diseases from 1950 to 2003 from the Jiangsu Provincial CDC and the Jiangsu Provincial Institute of Parasitic Diseases. Data from 1950 to 1990 included only monthly cases and fatalities at the county level, while data from 1991 to 2003 also indicated the age, sex, and occupation of cases. Data from 2004 to 2022 were collected from NIDRIS, which included sex, age, occupation, residential address, data of illness onset, data of diagnosis, and date of death.

Since 1950, data have been reported for so-called “class A” and “class B” infectious diseases, which are considered to be more severe. Since 2004, data have also been reported for “class C” notifiable infectious diseases, which are considered less severe.

No cases of plague, filariasis, hookworm disease, and forest encephalitis were reported in Jiangsu province during the study period, so they were not analysed in this study. Viral hepatitis was classified as subtype A, B, or non-A/non-B for data from 1990-1996, as A, B, C, or E or “unclassified” for data from 1997-2015, or as A, B, C, D, or E for data from 2016 onward. We analysed five subtypes of viral hepatitis, hepatitis A, B, C, D and E as individual diseases, and as aggregated data for traditional seasonal influenza and 2009 influenza A (H1N1) into influenza. The final analysis included 46 infectious diseases.

We divided diseases from classes A and B into the following categories, based on mode of transmission: intestinal, respiratory, vector-borne and zoonotic, sexually transmitted and blood-borne, except for neonatal tetanus transmitted by direct and indirect contact. Intestinal diseases included cholera, poliomyelitis, dysentery, typhoid/paratyphoid, viral hepatitis (before 1990), hepatitis A (after 1990), and hepatitis E (after 1997). Respiratory infectious diseases included smallpox, severe acute respiratory syndrome (SARS), influenza, measles, tuberculosis, epidemic cerebrospinal meningitis (ECM), pertussis, diphtheria, scarlet fever, avian influenza H7N9, avian influenza H5N1, and COVID-19. Vector-borne and zoonotic diseases included haemorrhagic fever with renal syndrome (HFRS), rabies, Japanese encephalitis, dengue fever, anthrax, brucellosis, leptospirosis, schistosomiasis, malaria, scrub typhus, typhus, and kala-azar. Sexually transmitted and blood-borne diseases included gonorrhoea, syphilis, acquired immune deficiency syndrome (AIDS), hepatitis B (after 1990), hepatitis C (after 1997), and hepatitis D (after 2016).

We divided the study period into four historical stages: stage I of rapid socio-economic development (1949-1965), stage II of stagnation (1966-1978), stage III of rapid growth (1979-2003), and stage IV of the after-SARS era (2004-2022).

### Socioeconomic data

Considering the availability, representativeness, and uniformity of indicators over the past 70 years, we characterised socio-economic development by using the number of medical and health institutions, and per capita GDP. We defined medical and health institutions as those established according to law to engage in disease diagnosis, treatment, and prevention, such as hospitals, clinics, outpatient departments, CDCs, and medical research institutes.

We obtained data on demographics, the annual number of medical and health institutions, and per capita GDP from the Jiangsu Provincial Statistical Data Compilation [12] for 1950-2005 and from the Jiangsu Provincial Bureau of Statistics [13] for 2005-2021.

### Statistical analysis

We defined annual incidence as the number of cases in one year divided by the population size in the same year, overall annual mortality as the number of deaths in one year divided by the total population size in the same year, and the annual fatality rate for a given infectious disease as the number of deaths attributed to that disease in one year divided by the number of cases of that disease in the same year.

We calculated the average annual percentage change (AAPC) for incidence and fatality rate and assessed the significance of changes using Student’s t*-*test. We described the trend as an “increase” or “decrease” if the slope of the change was associated with a two-sided *P* < 0.05, and as “stable” if it was not significant. We estimated 95% confidence intervals (CIs) for AAPCs. We further used joinpoint regression to examine incidence and fatality rate trends and explored correlations between the number of medical and health institutions or per capita GDP and the incidence or fatality rate using Pearson’s or Spearman’s correlation analysis, with significance set at *P* < 0.05.

We performed statistical analyses using R, version 3.5.3 (R core team, Auckland, New Zealand) and joinpoint regression using Joinpoint 4.9.0.1 (National Cancer Institute, Bethesda, Maryland, USA). We generated figures via GraphPad Prism 8.0. (GraphPad Software, San Diego, California, USA)

### Ethics statement

Informed consent and institutional ethics approval were unnecessary because this study was conducted using anonymised data from government databases, which collected data in accordance with relevant ethics and data protection guidelines.

## RESULTS

### Analysis across infectious disease categories

A total of 75 754 008 cases of notifiable infectious diseases were reported throughout the study period; malaria, schistosomiasis, influenza, dysentery, and hand-foot-mouth disease (HFMD) accounted for 81.85% of all cases (**Table 1**). The average annual incidence across all diseases and the entire study period was 1679.49 cases per 100 000 population, the average annual mortality was 3.05 deaths per 100 000 population, and the fatality rate was 1.82 deaths per 1000 cases. Rabies was associated with the highest fatality, followed by avian influenza H5N1, avian influenza H7N9, AIDS and neonatal tetanus.

**Table 1:** Incidence and fatality of 46 notifiable infectious diseases and their trends in Jiangsu province, China, 1950-2022

						Trend in incidence			Trend in case-fatality rate		
**Disease**	**Data period**	**Cases**	**Deaths**	**Annual incidence (cases per 100 000 persons)**	**Fatality rate (deaths per 1000 cases)**	**Trend**	**AAPC in % (95% CI)**	***P*-value**	**Trend**	**AAPC in % (95% CI)**	***P*-value**
Malaria	1950-2022	34681057	750	768.89	0.022	Stable	-9.9 (-44.1, 45.1)	0.67	Stable	-11.5 (-26.5, 6.5)	0.20
Schistosomiasis*	1951-2022	14068526	0	314.40	ND	Decrease	-20.1 (-25.9, -13.8)	<0.05	ND	ND	ND
Influenza	1958-2022	6141555	254	145.95	0.041	Stable	2.3 (-24.7, 39.1)	0.88	Stable	-7.1 (-28.7, 21.0)	0.59
Dysentery	1950-2022	5637834	4165	124.99	0.74	Decrease	-5.6 (-6.5, -4.7)	<0.05	Decrease	-9.7 (-15.3, -3.7)	<0.05
Measles	1950-2022	4406714	43647	97.70	9.91	Decrease	-12.0 (-16.5, -7.4)	<0.05	Decrease	-13.9 (-15.9, -11.8)	<0.05
Viral hepatitis	1960-2022	3401155	2813	82.49	0.83	Stable	0.1 (-2.9, 3.1)	0.97	Decrease	-3.2 (-6.2, -0.2)	<0.05
Hepatitis A	1990-2022	411220	132	17.18	0.32	Decrease	-14.5 (-17.4, -11.5)	<0.05	Decrease	-8.0 (-14.0, -1.6)	<0.05
Hepatitis B	1990-2022	536722	561	22.43	1.05	Stable	-1.9 (-4.3, 0.7)	0.15	Decrease	-9.3 (-12.5, -5.9)	<0.05
Hepatitis C	1997-2022	55596	33	2.78	0.59	Increase	11.2 (9.2, 13.2)	<0.05	Stable	-7.1 (-16.7, 3.6)	0.18
Hepatitis D	2016-2022	28	0	0.0049	0	Stable	17.6 (-22.6, 78.8)	0.36	Stable	0.0 (-0.0, 0.0)	ND
Hepatitis E	1997-2022	60637	49	3.03	0.81	Increase	10.0 (8.1, 12.0)	<0.05	Stable	0.9 (-17.2, 22.9)	0.93
HFMD	2008-2022	1478392	62	123.49	0.042	Stable	-0.3 (-13.7, 15.3)	0.97	Decrease	-33.8 (-54.7, -3.4)	<0.05
Pertussis	1955-2022	1096315	906	25.34	0.83	Decrease	-5.9 (-10.6, -0.8)	<0.05	Decrease	-13.4 (-18.0, -8.6)	<0.05
ECM	1950-2022	1067488	44776	23.67	41.95	Decrease	-9.1 (-11.1, -7.1)	<0.05	Decrease	-6.2 (-10.4, -1.8)	<0.05
Tuberculosis	1990-2022	887065	2567	35.79	2.89	Decrease	-2.9(-3.6, -2.1)	<0.05	Increase	20.5 (9.4, 32.8)	<0.05
Typhoid & paratyphoid	1950-2022	667161	1579	14.79	2.37	Decrease	-7.4 (-9.0, -5.7)	<0.05	Decrease	-14.3 (-16.1, -12.3)	<0.05
Syphilis	1990-2022	452238	40	18.25	0.088	Increase	25.9 (18.5, 33.8)	<0.05	Increase	19.3 (9.1, 30.3)	<0.05
Gonorrhea	1990-2022	446089	1	18.00	0.0022	Stable	0.2 (-4.2, 4.8)	0.94	Stable	-0.2 (-1.7, 1.3)	0.76
OID	2004-2022	304151	3	20.31	0.0099	Stable	0.2 (-4.9, 5.7)	0.93	Stable	-0.4 (-5.1, 4.5)	0.86
Japanese encephalitis	1950-2022	215428	15814	4.78	73.41	Decrease	-5.7 (-10.7, -0.3)	<0.05	Stable	-0.2 (-11.2, 12.3)	0.98
Diphtheria	1950-2022	209198	9447	4.64	45.16	Decrease	-14.3 (-17.5, -11.0)	<0.05	Decrease	-5.6 (-10.6, -0.3)	<0.05
Mumps	2004-2022	183852	1	12.28	0.0054	Stable	-3.4 (-6.8, 0.2)	0.06	Stable	0.0 (-9.2, 10.1)	1.00
Scarlet fever	1950-2022	136653	208	3.03	1.52	Stable	0.1 (-12.0, 14.0)	0.98	Decrease	-15.0 (-18.3, -11.5)	<0.05
HFRS	1955-2022	74300	2239	1.72	30.14	Stable	0.0 (-3.1, 3.1)	0.981	Stable	5.4(-1.6, 13.0)	0.13
Cholera	1950-2022	43776	144	0.97	3.29	Stable	3.2 (-14.9, 25.2)	0.75	Stable	1.1 (-3.7, 6.1)	0.67
Poliomyelitis	1955-2022	35423	669	0.82	18.89	Decrease	-7.9 (-13.4, -2.0)	<0.05	Stable	-3.5 (-24.6, 23.5)	0.78
COVID-19 ^†^	2020-2022	21740	0	8.68	0	ND	ND	ND	ND	ND	ND
AIDS	1990-2022	16862	2925	0.68	173.47	Increase	33.7 (19.6, 49.5)	<0.05	Stable	7.5 (-2.2, 18.2)	0.14
Kala-azar	1950-1978, 1989-2022	16491	26	0.42	1.58	Stable	-12.8 (-26.8, 3.8)	0.12	Decrease	-2.5 (-4.4, -0.5)	<0.05
Relapsing fever	1950-1989	12810	161	0.63	12.57	Decrease	-29.6 (-34.5, -24.4)	<0.05	Decrease	-10.6 (-11.1, -10.1)	<0.05
Leptospirosis	1962-2022	12413	12	0.31	0.97	Decrease	-7.8 (-13.9, -1.3)	<0.05	Stable	-3.2 (-6.6, 0.3)	0.07
Rubella ^‡^	2004-2022	10728	1	0.72	0.093	Decrease	-14.7 (-22.8, -5.7)	<0.05	Increase	35.7 (1.0, 82.4)	<0.05
AHC	2004-2022	10138	0	0.68	0	Stable	1.7 (-13.2, 19.2)	0.83	Stable	0.0 (-0.0, -0.0)	ND
Typhus	1950-2022	5802	157	0.13	27.06	Decrease	-14.8 (-19.0, -10.5)	<0.05	Decrease	-11.7 (-13.9, -9.5)	<0.05
Smallpox	1950-1989	4895	333	0.25	68.03	Decrease	-32.0 (-38.8, -24.4)	<0.05	Decrease	-14.7 (-17.9, -11.4)	<0.05
Rabies	1955-2022	3926	3687	0.091	939.12	Decrease	-3.9 (-7.1, -0.6)	<0.05	Stable	2.1 (-6.9, 11.9)	0.66
Brucellosis	1955-2022	1628	0	0.038	0	Increase	12.0 (3.3, 21.6)	<0.05	Stable	0.0 (-0.0, 0.0)	ND
Scrub typhus	1955-1989	635	0	0.034	0	Increase	39.7 (24.5, 56.8)	<0.05	Stable	0.0 (-0.0, 0.0)	ND
Neonatal tetanus	1990-2022	532	78	0.021	146.62	Stable	5.7 (-27.2, 53.4)	0.77	Stable	4.4 (-20.8, 37.8)	0.76
Dengue	1990-2022	380	0	0.015	0	Stable	-4.1 (-21.6, 17.4)	0.69	Stable	0.0 (-0.0, 0.0)	ND
Avian influenza H7N9	2013-2022	225	95	0.028	422.22	Decrease	-52.2 (-73.0, -15.2)	<0.05	Stable	-39.7 (-66.9, 10.0)	0.09
Leprosy	2004-2022	184	0	0.012	0	Decrease	-17.0 (-26.7, -5.9)	<0.05	Stable	0.0 (-0.0, 0.0)	ND
Anthrax	1955-2022	175	6	0.0040	34.29	Stable	-6.1 (-30.8, 27.4)	0.69	Decrease	-0.7 (-1.3, -0.1)	<0.05
Echinococcosis	2004-2022	65	0	0.0043	0	Stable	1.5 (-4.3, 7.5)	0.61	Stable	0.0 (-0.0, 0.0)	ND
SARS	2003-2022	6	0	0.00038	0	Decrease	-23.8 (-24.1, -23.5)	<0.05	Stable	0.0 (-0.0, 0.0)	ND
Avian influenza H5N1	2004-2022	3	2	0.00020	666.67	Stable	-6.4 (-14.4, 2.3)	0.14	Stable	-2.9 (-15.8, 11.8)	0.66

AAPC – average annual percentage change, AHC – acute haemorrhagic conjunctivitis, AIDS – acquired immune deficiency syndrome, CI – confidence interval, ECM – epidemic cerebrospinal meningitis, HFRS – haemorrhagic fever with renal syndrome, HFMD – hand-foot-mouth disease, JE – Japanese encephalitis, ND – not done, OID – infectious diarrhoeal diseases other than cholera, dysentery, or typhoid/paratyphoid, SARS – severe acute respiratory syndrome

*Data on deaths due to schistosomiasis before 2004 were unavailable.

†Annual incidence data for COVID-19 were available for only three years, so no joinpoint regression could be performed.

‡Only one death in rubella occurred in 2022, leading to an increasing trend in fatality rate.

**Figure 2:**
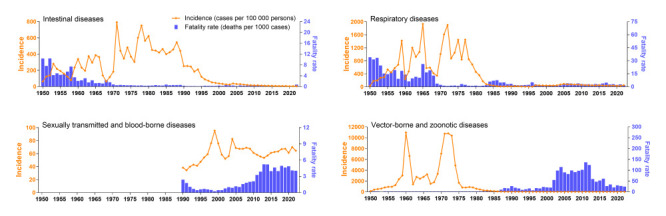
Incidence and fatality rates for notifiable infectious diseases in classes A-B in Jiangsu province, China, stratified by transmission route.

The incidence of diseases in classes A-B decreased during the study period, with an AAPC of -2.1% (95% CI = -4.0, -0.3). In contrast, the incidence of class C diseases increased from 2004 to 2022, with an AAPC of 10.8% (95% CI = 4.9, 17.0). The fatality rate remained stable for diseases in classes A-B (AAPC = -1.7%; 95% CI = -6.5, 3.4), and class C (AAPC = 16.6%, 95% CI = -9.9, 50.9). Joinpoint regression of diseases in classes A-B during the study period showed a decreasing trend in incidence and fatality rate for intestinal diseases, a decreasing trend in incidence but increasing trend in fatality rate for vector-borne and zoonotic diseases, and an increasing trend in incidence for sexually transmitted and blood-borne diseases (**Table 2**, **Figure 2**).

**Table 2:** Incidence and fatality of notifiable infectious diseases (classes A-B) and their trends in Jiangsu province, China, 1950-2022, stratified by transmission route and historical period

	Incidence					Fatality				
	**Cases**	**Annual incidence (cases per 100 000 persons)**	**Trend**	**AAPC in % (95% CI)**	***P*-value**	**Deaths**	**Fatality rate (deaths per 1000 cases)**	**Trend**	**AAPC in % (95% CI)**	***P*-value**
**Disease category**										
Intestinal	8 726 868	193.48	Decrease	-4.4 (-5.9, -3.0)	<0.05	8518	0.98	Decrease	-4.4 (-5.8, -3.0)	<0.05
Respiratory	1 371 7817	304.13	Stable	-2.3 (-8.5, 4.3)	0.49	102175	7.45	Stable	-3.1 (-8.5, 2.6)	0.28
Sexually transmitted and blood-borne	1 507 535	33.42	Increase	1.8 (0.9, 2.8)	<0.05	3560	2.36	Stable	1.7 (-1.1, 4.6)	0.23
Vector-borne and zoonotic	49 093 542	1088.42	Decrease	-8.1 (-10.0, -6.1)	<0.05	22852	0.47	Increase	3.5 (0.1, 7.0)	<0.05
**Socioeconomic Period**										
Stage I (1949-65)	22 174 371	3397.34	Increase	16.4 (6.8, 26.8)	<0.05	85970	3.88	Stable	-3.5 (-19.2, 15.2)	0.69
Stage II (1966-78)	40 762 201	5847.67	Stable	-5.8 (-30.1, 27.2)	0.70	33873	0.83	Stable	-9.9 (-25.6, 9.1)	0.28
Stage III (1979-2003)	8 534 292	513.16	Decrease	-11.0 (-14.2, -7.7)	<0.05	10563	1.24	Increase	4.3 (0.2, 8.6)	<0.05
Stage IV (2004-22)	2 041 585	136.32	Decrease	-3.5 (-4.4, -2.6)	<0.05	7035	3.45	Stable	1.1 (-0.8, 3.0)	0.26
Entire study period	73 512 449	1629.79	Decrease	-2.1(-4.0, -0.3)	<0.05	137441	1.87	Stable	-1.7 (-6.5, 3.4)	0.51

AAPC – average annual percentage change, CI – confidence interval

**Figure 3:**
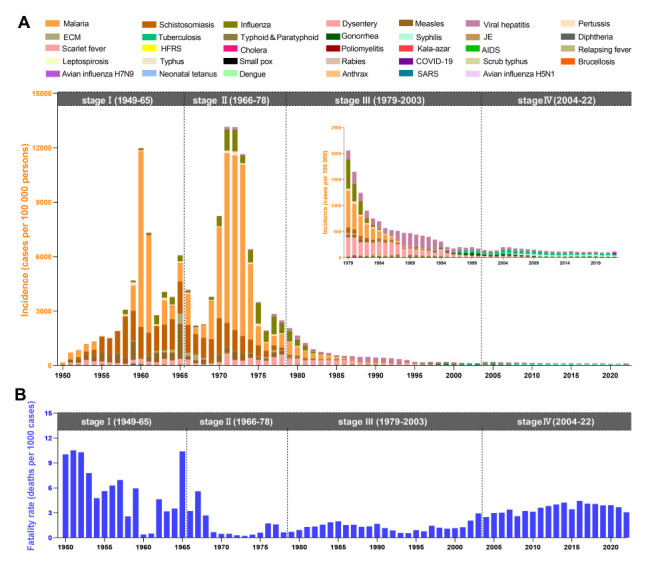
Incidence and fatality rates for notifiable infectious diseases in classes A-B in Jiangsu province, China, stratified by historical period. **Panel A.** Incidence for notifiable infectious diseases in classes A-B, according to historical period. **Panel B.** Fatality rates for notifiable infectious diseases in classes A-B, according to historical period.

Across all diseases in classes A-B, the annual incidence showed a shallow inverted U-shape during the historical period of rapid socioeconomic development, a deep inverted U-shape during stagnation, a stable low level during rapid growth, and a rapid decline in the post-SARS era (**Table 2** and **Figure 3**, Panel A). The incidence increased in the first stage, stabilised in the second stage, and decreased in the third and fourth stages. The fatality rate decreased from 3.88 to 0.83 deaths per 1000 cases from the first to third stage, returning to 3.45 deaths per 1000 cases in the fourth stage (**Table 2** and **Figure 3**, Panel B). In parallel, the fatality rate was stable in the first, second and fourth stages, but showed an increasing trend in the third stage.

**Figure 4:**
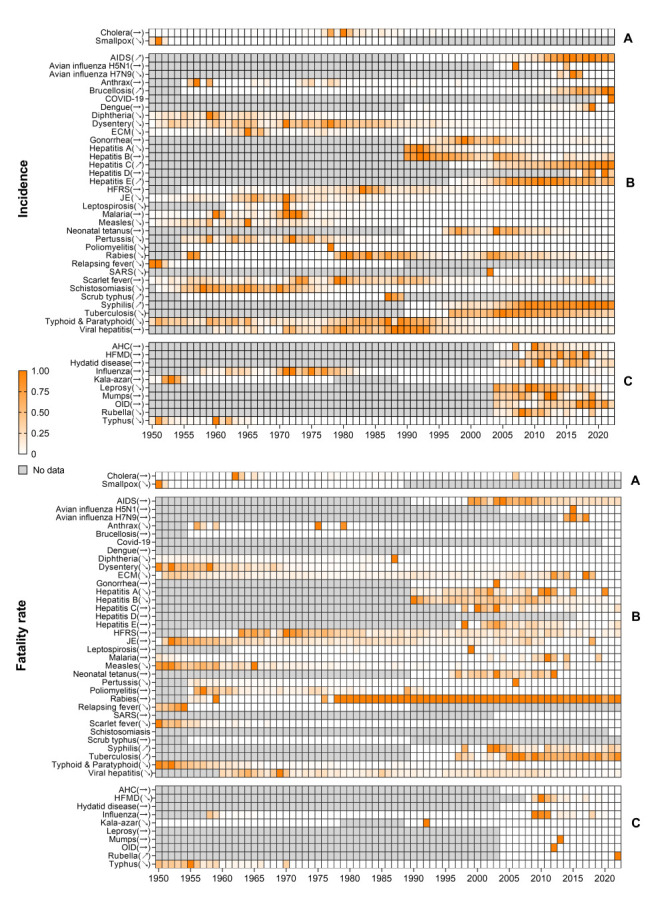
Trends in the incidence and fatality rates of notifiable infectious diseases in classes A-B in Jiangsu province, China, 1950-2022. Infectious diseases are grouped according to the latest classification of Class A, B and C, then alphabetical order. The incidence/fatality rate for each infectious disease was standardised from 0 to 1 according to percentile rank, and represented by the color scale (from 0 to 1; where 1 is the highest rate and 0 is the lowest). Gray represents no data. Trend arrows next to the names of diseases indicate whether the annual average percentage change is positive and statistically significant (upward arrow), negative and significant (downward arrow), or not significant (horizontal arrow).

From 2004 to 2022, the annual incidence of infectious diseases in classes A-B and the rate of associated fatality in Jiangsu province correlated positively with the corresponding statistics at the national level (**Figure 4**), suggesting our analysis may be representative of the national situation.

### Analysis of infectious diseases individually

**Figure 5:**
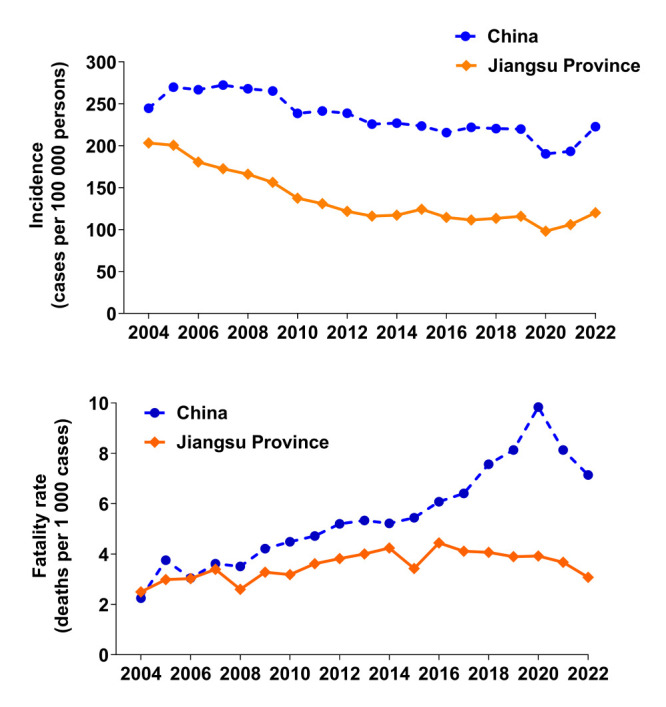
Comparison of incidence of notifiable infectious diseases (classes A-B) and associated fatality between Jiangsu province (orange) and all of China (blue), 2004-2022.

Analysis of individual diseases revealed a decreasing trend in the incidence of the following 20 diseases (**Table 1**, **Figure 5**): avian influenza H7N9, diphtheria, dysentery, ECM, hepatitis A, Japanese encephalitis, leprosy, leptospirosis, measles, pertussis, poliomyelitis, rabies, relapsing fever, rubella, SARS, schistosomiasis, smallpox, tuberculosis, typhoid/paratyphoid, and typhus. For a partially overlapping set of 16 diseases, the rate of associated fatalities showed a decreasing trend (**Table 1**, **Figure 5**): anthrax, diphtheria, dysentery, ECM, HFMD, hepatitis A, hepatitis B, kala-azar, measles, pertussis, relapsing fever, scarlet fever, smallpox, typhoid/paratyphoid, typhus, and viral hepatitis.

Conversely, the analysis showed an increasing trend in the incidence of AIDS, brucellosis, hepatitis C, hepatitis E, scrub typhus, and syphilis, as well as an increasing trend in the rate of fatalities due to rubella, syphilis, and tuberculosis (**Table 1**, **Figure 5**).

The five most frequent diseases in the first and second historical stages were malaria, schistosomiasis, measles, influenza, and dysentery. In the third stage, viral hepatitis ranked first, followed by dysentery, malaria, influenza, and typhoid/paratyphoid. HFMD was the most frequent in the fourth stage, followed by tuberculosis, viral hepatitis, syphilis, and infectious diarrheal diseases other than cholera, dysentery, and typhoid and paratyphoid (OID) (**Table 3**).

**Table 3:** Incidence rank of notifiable infectious diseases in Jiangsu province, China, stratified by historical period

	Stage I (1949-1965)		Stage I (1966-1978)		Stage III (1979-2003)		Stage IV (2004-2022)	
**Ranking**	**Disease**	**Annual incidence (cases per 100 000 persons)**	**Disease**	**Annual incidence (cases per 100 000 persons)**	**Disease**	**Annual incidence (cases per 100 000 persons)**	**Disease**	**Annual incidence (cases per 100 000 persons)**	
1	Malaria	1373.78	Malaria	3481.56	Viral hepatitis	137.04	HFMD	123.49	
2	Schistosomiasis	1169.53	Schistosomiasis	944.63	Dysentery	136.55	Tuberculosis	45.44	
3	Measles	389.58	Influenza	604.68	Malaria	86.52	Viral hepatitis	31.57	
4	Influenza	255.13	Dysentery	296.30	Influenza	46.83	Syphilis	27.81	
5	Dysentery	179.14	Measles	233.65	Typhoid & paratyphoid	26.86	OID	20.31	

HFMD – hand-foot-mouth disease, OID – infectious diarrhoeal diseases other cholera

### Associations of incidence or fatality rate with socioeconomic indicators

The number of medical and health institutions correlated negatively with an annual incidence of infectious disease across the study period, but not with the rate of associated fatalities; some of these correlations alternated between negative or positive across the four historical periods (**Table 4**). We observed similar results for GDP per capita.

**Table 4:** Correlations of incidence of notifiable infectious diseases (classes A-B) and associated fatality in Jiangsu with the annual number of medical and health institutions or per capita gross domestic product, stratified by historical period

		Stage I (1949-65)		Stage II (1966-78)		Stage III (1979-2003)		Stage IV (2004-21)		Entire study period	
**Disease indicator**	**Socioeconomic indicator**	**r**	***P*-value**	**r**	***P*-value**	**r**	***P*-value**	**r**	***P*-value**	**r**	***P*-value**
Incidence	Annual number of hospitals and clinics	0.92	<0.05	-0.68	<0.05	-0.87	<0.05	-0.89	<0.05	-0.69	<0.05
	Per capita GDP	0.90	<0.05	-0.06	0.86	-0.99	<0.05	-0.97	<0.05	-0.90	<0.05
Fatality rate	Annual number of medical and health institutions	-0.70	<0.05	0.71	<0.05	-0.05	0.81	0.78	<0.05	0.08	0.51
	Per capita GDP	-0.37	0.19	-0.35	0.24	-0.02	0.92	0.76	<0.05	0.05	0.69

GDP – gross domestic product

## DISCUSSION

Our analysis of the Jiangsu province for the 1950-2022 period suggests that public health policies have been effective at eradicating or reducing the incidence of numerous infectious diseases and the associated fatality, but that the incidence of sexually transmitted and blood-borne diseases, mainly syphilis and AIDS, has increased. Our findings provide support for past public health interventions, while indicating the need to strengthen or redesign future interventions to control reemerging threats.

We observed strong decreases in the incidence of intestinal, vector-borne, and zoonotic diseases during the study period. Several factors may help explain these decreases. The Patriotic Health Movement, launched in the 1950s, promoted sanitation and public health measures to eradicate vector-borne diseases through interventions such as immunisation, access to toilets and clean drinking water, and health education [14,15]. The immunisation campaign that began in the 1950s led to the eradication of smallpox from the country and the near-eradication of poliomyelitis, measles, ECM, pertussis, and Japanese encephalitis for different age groups [16]. Another factor is the increase in GDP per capita since 1950, which has given many people greater access to health, education, and other services. Indeed, we found that it correlated negatively with the annual incidence of infectious disease, consistent with previous observations in China and other countries that better economic conditions correlate with a lower incidence of infectious disease [17,18]. This finding can help inform policymakers of the role of economic fluctuations in managing pandemics. Further, it may provide guidance on the future impact of macroeconomic downturns on the spread of infectious diseases. Strategies to identify and engage high-risk groups of infectious diseases, while also safeguarding potentially tight budgets, should be prioritised for the health sector, as infectious diseases, like economic crises, are difficult to control once they begin to spread.

In contrast to the decline in intestinal, vector-borne, and zoonotic diseases that we observed, we found an increase in the annual incidence of sexually transmitted and blood-borne diseases, mainly syphilis and AIDS. China’s efforts to end the commercial sex industry in the 1950s and 1960s through the closure of brothels, the treatment of sex workers with penicillin, and regular syphilis testing among former sex workers helped with controlling sexually transmitted diseases (STDs) [19,20] and, alongside other measures, lead to their near elimination in the country [21], only for their incidence to increase again with “reform and opening up” in the 1980s [22]. The ensuing social transformation generated large numbers of men with disposable income, women who earn money through sexual intercourse [23], men who have intercourse with men [24], drug users, and migrant workers [25], all of whom are at higher risk of sexually transmitted diseases than the general population. These diseases can then spread from high-risk populations into the general community.

We found a decreasing trend in fatality rates associated with intestinal diseases, likely reflecting the widespread availability of antibiotic therapy for bacterial infections such as dysentery and typhoid/paratyphoid [26,27], which were the most frequent intestinal diseases during our study period.

We found that the number of medical and health institutions and GDP per capita correlated with incidence or fatality rates of infectious diseases negatively or positively depending on the historical period. This may reflect the evolution of public health policies and infrastructure during China’s development. In the rapid socioeconomic development stage (1949-1965), the number of medical and health institutions correlated positively with annual incidence, but negatively with fatality rates. This likely reflects improvements in disease reporting and the success of programmes to improve rural health and prevent disease that started after the first National Health Congress in August 1950 and continued until the 1980s [14]. As part of these programmes, “epidemic prevention stations” were set up around the country based on similar stations in the Soviet Union. By 1965, approximately 2500 epidemic prevention stations were operating across more than two-thirds of Chinese counties [14]. In parallel, the abovementioned Patriotic Health Movement mobilised local governments and communities to improve nutrition, sanitation, water quality, and control of infectious disease [14,15]. Between 1950 and 1952, over 512 million of the country’s population of 600 million were vaccinated against smallpox, and the last smallpox outbreak in China occurred in 1960, already 20 years before it was eradicated globally.

During the socioeconomic stagnation stage (1966-1978), the number of medical and health institutions correlated negatively with annual incidence but positively with fatality rate, which may reflect that Chinese efforts to control infectious diseases stagnated. During this period, many epidemic prevention stations were forced to close. Mass migration of people between urban and rural areas led to a resurgence of infectious diseases, particularly parasitic ones, that had been controlled during the previous historical period.

In the subsequent rapid growth (1979-2003) and post-SARS (2004-2022) stages, the number of medical and health institutions and GDP per capita correlated negatively with both annual incidence and fatality rate, suggesting further improvement of medical and health services in parallel with economic growth. Starting with reform and opening up in 1979, the national government addressed the severe shortage of medical and health resources and low efficiency of service provision by opening public hospitals and, after 2001, permitting the construction of private ones [28]. The country established a nationwide Expanded Programme on Immunisation based on a cold-chain vaccine system recommended by the World Health Organisation [29]. After the 2003 SARS outbreak highlighted inadequacies in China's infectious disease prevention system [30], the country created a network of Centers for Disease Control modelled on the US system, and that network set up NIDRIS in 2004. Through NIDRIS, cases of notifiable infectious diseases and associated deaths are reported by hospitals directly and electronically to the Chinese Centers for Disease Control, improving the timeliness and completeness of case detection.

We found an unexpected trend of increasing fatality rates due to infectious disease in the rapid growth stage (1979-2003). This may reflect the expansion and improvement of Disease Surveillance Points across the country, through which causes of death are reported and analysed [31,32].

Malaria was the most frequent notifiable infectious disease in Jiangsu province across the whole study period, while HFMD was the fifth most frequent. This ranking varied across the four historical stages. Viral hepatitis replaced malaria as the most frequent disease during the rapid growth stage (1979-2003), and HFMD replaced viral hepatitis during the post-SARS stage. These findings are consistent with national monitoring data [11,33].

Throughout our study period, the highest fatality rates were associated with three zoonoses: rabies (for which fatality rates worldwide are virtually 100%), avian influenza H5N1, and avian influenza H7N9. The fatality rates associated with avian influenza may be overestimated because of the potentially numerous asymptomatic or mild cases that go unreported. Passage of influenza virus from birds to humans remains a public health threat because of the lack of obvious symptoms in birds, but the virus may become less virulent if it begins to circulate widely in humans [34,35].

Our findings should be interpreted with caution due to several limitations. We cannot be sure that all cases of disease and associated mortality were reported, especially early during the observation period, before reporting systems were digitised. The available data did not include all infectious diseases relevant to China’s public health situation, such as severe fever with thrombocytopenia syndrome, of which local outbreaks have been reported [36], or scrub typhus, which was removed from the list of notifiable infectious diseases in 1989 but which recently caused outbreaks in southern China [37]. Third, we were unable to repeat our analyses at the national level because of restrictions on access to such data. While our analysis of Jiangsu aligns with national reports, whether our data from this province can be extrapolated to the rest of the country remains to be investigated.

## CONCLUSIONS

Despite these limitations, our work provides what appears to be the longest retrospective analysis of notifiable infectious diseases in any part of China. During the period from 1950 to 2022, China has succeeded in strongly reducing the incidence of numerous infectious diseases and associated mortality, in part through economic growth and sustainable investment in health systems. At the same time, the growing prosperity has challenged the control of other infectious diseases, primarily sexually transmitted and blood-borne ones. These insights from Jiangsu province may help guide the further improvement of public health policy and infrastructure in China and other countries that struggle to control infectious disease in the face of socioeconomic transformation.
